# Prediction of Severe Baseline Asymptomatic Carotid Stenosis and Subsequent Risk of Stroke and Cardiovascular Disease

**DOI:** 10.1161/STROKEAHA.123.046894

**Published:** 2024-09-25

**Authors:** Michiel H.F. Poorthuis, Steven H.J. Hageman, Aernoud T.L. Fiolet, L. Jaap Kappelle, Michiel L. Bots, Ph. Gabriel Steg, Frank L.J. Visseren, Deepak L. Bhatt, Gert J. de Borst

**Affiliations:** 1 Department of Neurology (M.H.F.P., L.J.K.), University Medical Center Utrecht, Utrecht University, the Netherlands.; 2 Department of Vascular Medicine (S.H.J.H., F.L.J.V.), University Medical Center Utrecht, Utrecht University, the Netherlands.; 3 Department of Cardiology (A.T.L.F.), University Medical Center Utrecht, Utrecht University, the Netherlands.; 4 Julius Center for Health Sciences and Primary Care (M.L.B.), University Medical Center Utrecht, Utrecht University, the Netherlands.; 5 Department of Vascular Surgery (G.J.B.), University Medical Center Utrecht, Utrecht University, the Netherlands.; 6 Université Paris-Cité, INSERM-UMR1148, F-75018, Assistance Publique-Hôpitaux de Paris, Hôpital Bichat, French Alliance for Cardiovascular Trials, France (P.G.S.).; 7 Institut Universitaire de France, Paris (P.G.S.).; 8 Mount Sinai Fuster Heart Hospital, Icahn School of Medicine at Mount Sinai, New York, NY (D.L.B.).

**Keywords:** asymptomatic diseases, cardiovascular diseases, carotid stenosis, epidemiologic methods, mass screening, risk assessment, stroke

## Abstract

**BACKGROUND:**

Risk models to identify patients at high risk of asymptomatic carotid artery stenosis (ACAS) can help in selecting patients for screening, but long-term outcomes in these patients are unknown. We assessed the diagnostic and prognostic value of the previously published Prevalence of ACAS (PACAS) risk model to detect ACAS at baseline and to predict subsequent risk of stroke and cardiovascular disease (CVD) during follow-up.

**METHODS:**

We validated the discrimination and calibration of the PACAS risk model to detect severe (≥70% narrowing) ACAS with patients from the Reduction of Atherothrombosis for Continued Health registry. We subsequently calculated the incidence rates of stroke and CVD (fatal and nonfatal stroke or myocardial infarction or vascular death) during follow-up in 4 risk groups (low, medium, high, and very high, corresponding to sum scores of ≤9, 10–13, 14–17, and ≥18, respectively).

**RESULTS:**

Among 26 384 patients, aged between 45 and 80 years, without prior carotid procedures, 1662 (6.3%) had severe baseline ACAS. During ≈70 000 patient-years of follow-up, 1124 strokes and 2484 CVD events occurred. Discrimination of the PACAS model was 0.67 (95% CI, 0.65–0.68), and calibration showed adequate concordance between predicted and observed risks of severe baseline ACAS after recalibration. Significantly higher incidence rates of stroke (*P*_trend_<0.011) and CVD (*P*_trend_<0.0001) during follow-up were found with increasing PACAS risk groups. Among patients with high PACAS sum score of ≥14 (corresponding to 27.7% of all patients), severe baseline ACAS prevalence was 11.4%. In addition, 56.6% of incident strokes and 64.9% of incident CVD events occurred in this group.

**CONCLUSIONS:**

The PACAS risk model can reliably identify patients at high risk of severe baseline ACAS. Incidence rates of stroke and CVD during follow-up were significantly higher in patients with high PACAS sum scores. Selective screening of patients with high PACAS sum scores may help to prevent future stroke or CVD.

Patients with established cardiovascular disease (CVD) are at high risk of recurrent events. Screening for precursors of CVD in other vascular bed, such as asymptomatic carotid artery stenosis (ACAS), is not recommended in the current guidelines.^1^ ACAS is associated with a substantial risk of ischemic stroke and myocardial infarction and might, therefore, be used as a target for screening.^2,3^ Early detection of ACAS enables prevention strategies to reduce the incidence of stroke and CVD in individuals by optimizing modifiable risk factors. Lifestyle interventions and medical preventive therapy, including weight reduction, smoking cessation, antihypertensive therapy, lipid-lowering therapy, and management of diabetes are recommended to prevent avoidable disability and premature deaths. Guidelines recommend considering antiplatelet therapy, but, in practice, most patients will receive antithrombotic therapy because ACAS is often detected in patients with known CVD.^4,5^

**See related article, p 2641**

Population-level screening for ACAS or selection of individuals for screening based on age or sex alone is not cost-effective because of the low overall prevalence.^6,7^ Risk models combining multiple predictors are able to identify groups of individuals at high risk of ACAS and may be used to select individuals for selective screening.^8,9^ The recently developed Prevalence of ACAS (PACAS) risk model was derived from a preclinical setting of 0.6M individuals attending screening clinics and showed reliable predictions of the prevalence of ACAS.^10^ However, it has not been validated to predict severe baseline ACAS in a clinical population of patients with established atherothrombotic CVD or multiple atherothrombotic risk factors.^10–14^ In addition, it is unclear whether patients with higher PACAS sum scores are also at higher risk of stroke and CVD during follow-up.^15^ We aimed to use the PACAS risk model to inform patients about their individual risk and to prioritize appropriate patients for treatment to prevent stroke and CVD.

We validated the PACAS risk model in the large population of the REACH registry (Reduction of Atherothrombosis for Continued Health) to assess its role for selective screening. We then calculated the risk of stroke and CVD during follow-up according to different risk groups of PACAS sum scores to assess whether patients selected for selective screening are also at high risk of major adverse cardiovascular events.

## METHODS

Data not published within the article are available from the REACH authors upon reasonable request.

### External Validation Cohort

Data of patients who were prospectively included in the REACH registry were used. The design of the REACH registry has been reported in detail previously.^16^ In summary, the REACH registry is an international, prospective, observational registry that recruited patients between 2003 and 2004, aged ≥45 years, from 5000 sites in 44 countries with documented symptomatic coronary artery disease (angina, myocardial infarction, or angioplasty/stent/bypass), cerebrovascular disease (ischemic stroke or transient ischemic attack [TIA]), peripheral artery disease (historical or current intermittent claudication associated with an ankle-brachial index <0.9), or at least 3 predefined atherothrombotic risk factors. Patients already enrolled in a clinical trial, hospitalized patients, or those who might have difficulty appearing for a follow-up visit were excluded. Patients were evaluated at baseline for a range of demographic, medical (among which the status of ACAS), and laboratory characteristics, before being reevaluated annually for up to 48 months post-baseline to ascertain whether they experienced any clinical events or hospitalizations. Final database lock was in April 2009. For the current study, patients outside the age range of 45 to 80 years (n=8402), with prior carotid interventions (n=4520), without baseline or follow-up date registered (n=273), or in whom the status of ACAS was unknown (n=25 844) were excluded (Figure S1). The study design was approved by the institutional review board in each participating country. All patients in the REACH registry gave written informed consent.

### Outcomes

The PACAS risk model was validated using severe baseline ACAS as predicted outcome, defined as reported narrowing of the carotid artery of ≥70%. We also calculated the incidences of (1) fatal or nonfatal stroke and (2) CVD events, that is, the composite of nonfatal or fatal stroke or myocardial infarction, or vascular death, during follow-up.

### Statistical Analysis

The continuous predictor systolic blood pressure was truncated at 70 and 240 mm Hg to limit the effect of outliers. The percentage of participants with missing data was low for most predictors (Table S1). Missing data on predictors were imputed with chained equations, and we created 10 imputed data sets with 200 iterations.

#### External Validation of the PACAS Risk Score to Detect Severe Baseline ACAS

The original regression formula (using the β-coefficients of the original logistic regression equation of ≥70% baseline ACAS) of the PACAS risk model was applied to each individual of the REACH registry to calculate the probability of severe baseline ACAS. We also calculated the PACAS sum score for each patient (see Table 1 for the risk scores assigned to each predictor).^10^ The PACAS model includes age, sex, and vascular risk factors (current smoking, diabetes, history of stroke or TIA, coronary artery disease, peripheral artery disease, blood pressure, and blood lipids) as predictors.^10^ HDL (high-density lipoprotein) cholesterol to calculate total cholesterol was not available in the REACH registry, and sum score, therefore, ranged from 0 to 24. We examined the predictive performance of discrimination and calibration. Discrimination quantifies the ability of the risk model to distinguish patients with and without severe baseline ACAS and was assessed using the area under the receiver operating characteristic (AUROC) curve. We calculated the AUROC curve per imputed data set, and we used Rubin rules to pool the AUROC curves. We estimated the mean probability for each patient across the 10 imputed data sets, and we split the predicted risks in 10 groups and plotted mean predicted and observed risks of severe baseline ACAS with corresponding 95% CIs for each decile in a calibration plot. We assessed calibration with calibration slope and calibration in the large. We calculated discrimination and calibration in all patients and in men and women separately.

**Table 1.**
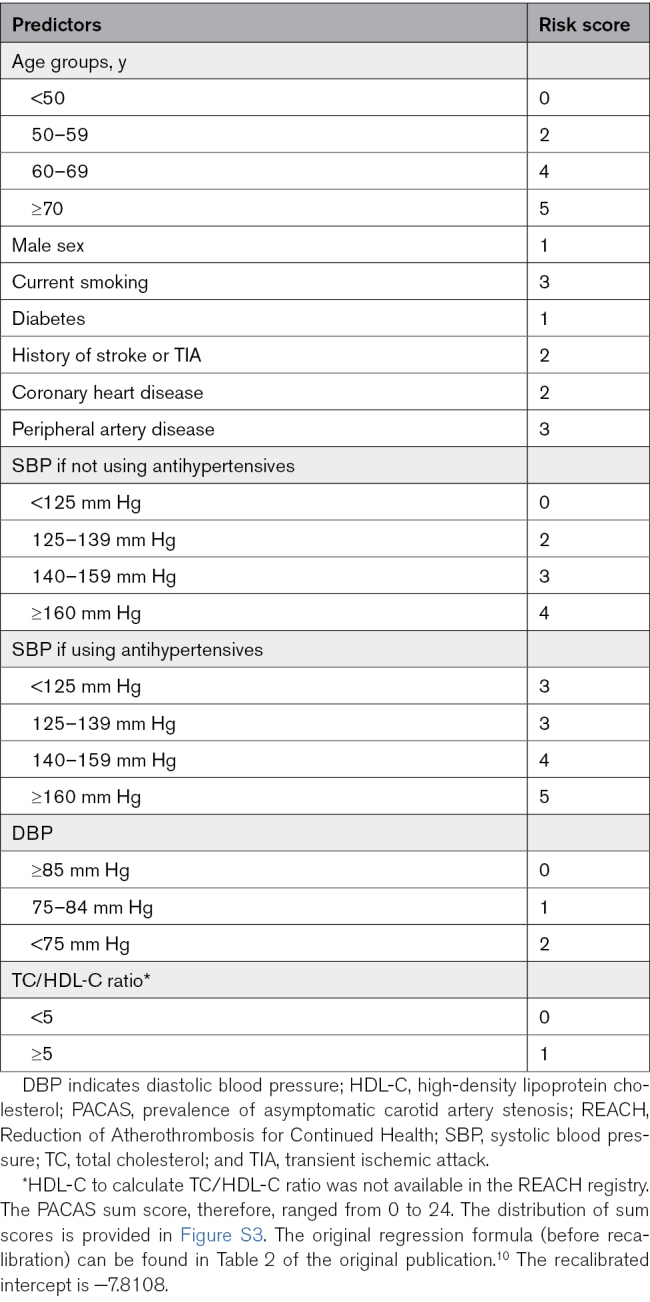
The PACAS Risk Score^10^

We recalibrated the intercept of the regression formula to the prevalence of ≥70% baseline ACAS in the validation cohort by fitting a logistic model with a fixed calibration slope and the intercept as the only free parameter (recalibration in the large).^17^

#### Clinical Utility of the PACAS Risk Score

We assessed the clinical utility of the PACAS score for selective screening by calculating screening characteristics (prevalence, number needed to screen [NNS] to detect 1 patient with severe ACAS, sensitivity, specificity, and positive and negative predictive values) for sum scores ≥14 and ≥18. These could serve as possible cutoff values to select individuals for selective screening.

#### Prognostic Significance of PACAS Risk Model to Predict Stroke and CVD During Follow-Up

We subsequently calculated incidence rates of stroke and CVD during follow-up for 4 risk groups (using arbitrary cutoffs of sum scores: 0–9, 10–13, 14–17, and 18–24). Cox proportional hazards models with the risk groups as the only covariate were used to estimate hazard ratios and 95% CIs for fatal or nonfatal stroke and the composite CVD during follow-up. For comparison of risk groups, the variance of the log hazards in each group was calculated from the variances and covariances of the log hazard ratios. This provides group-specific CIs that allow comparison between risk groups without the choice of a reference group.^18,19^ Incidence rates of stroke and CVD in patients with and without severe baseline ACAS were compared with incidence rate ratios. Patients were censored at nonvascular death for the outcome CVD or all-cause mortality for the outcome stroke. Patients were also censored at the time of carotid interventions (surgery or stenting) if performed during follow-up.

STATA, version 15.1, was used for all statistical analyses, and R, version 3.5.1, was used for constructing the figures. For external validation, our study adhered to the Transparent Reporting of a Multivariable Prediction Model for Individual Prognosis or Diagnosis statement (Table S2).^20^

## RESULTS

**Figure 1.**
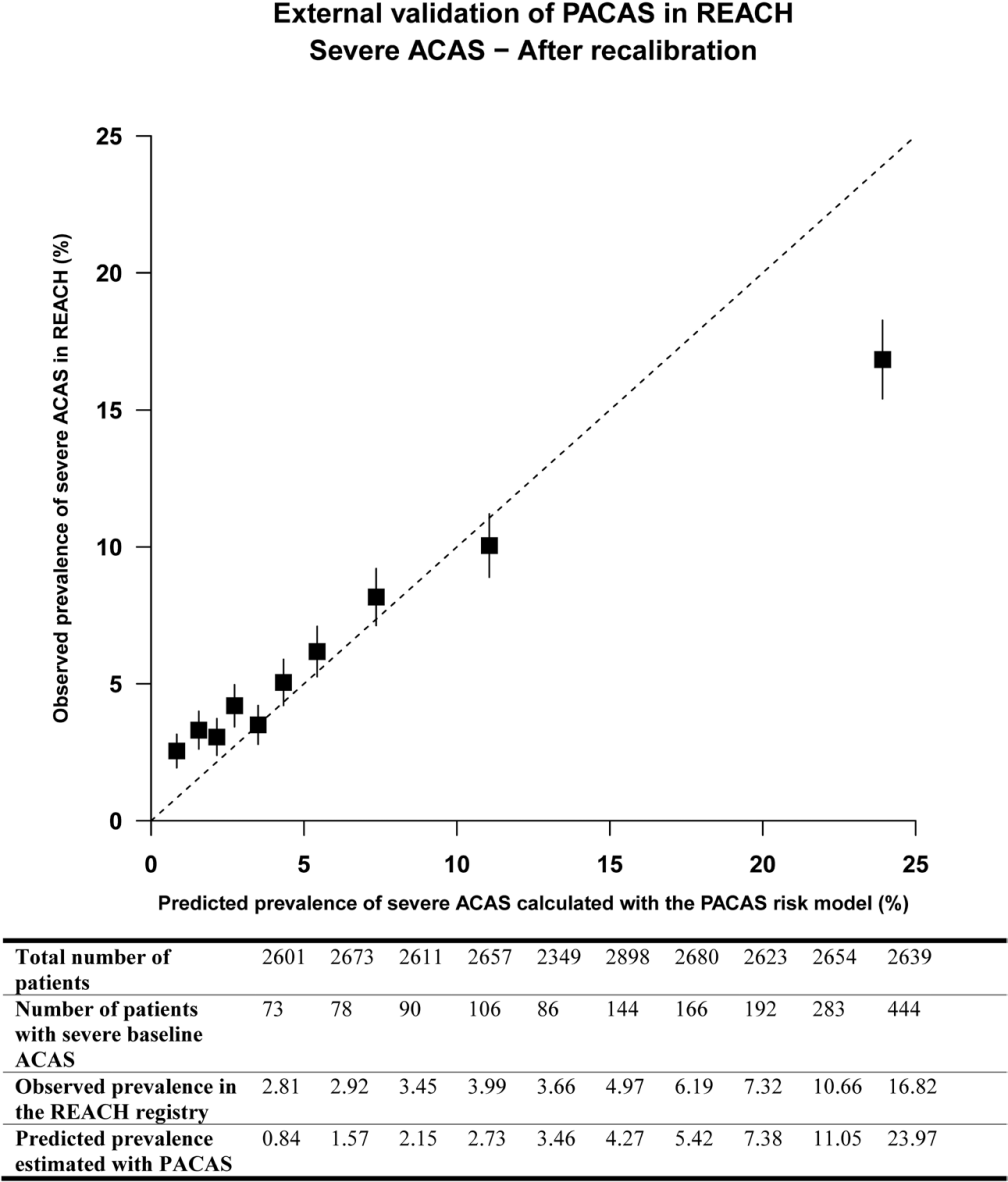
**Calibration plot of external validation of prevalence of asymptomatic carotid artery stenosis (PACAS) in the REACH registry (Reduction of Atherothrombosis for Continued Health).** The calibration plot showing the mean predicted risk against the observed risk of severe baseline asymptomatic carotid artery stenosis (ACAS) across deciles of predicted risk. The boxes represent the mean predicted risk for each decile, and the vertical lines represent the 95% CIs. The dotted diagonal line indicates perfect calibration. Boxes above the diagonal line indicate underestimation of risk and boxes below the diagonal line, overestimation of risk. The mean calibration (calibration in the large) was 0, indicating adequate overall predictions, and the calibration slope was 0.618, suggesting that estimated risks are too extreme, that is, too high for patients who are at high risk and too low for patients who are at low risk.

Baseline characteristics of the 26 384 patients are shown in Table 2. Severe baseline ACAS was present in 1662 (6.3%) patients. The distribution of PACAS sum scores is shown in Figure S2. Discriminative performance of the PACAS point chart, as assessed by AUROC curve, was 0.65 (95% CI, 0.64–0.67). This was slightly higher using the risk equation, with an AUROC curve of 0.67 (95% CI, 0.65–0.68). The original model underestimated the risk of severe baseline ACAS in the REACH registry (Figure S3), but after recalibration (with adjusting the intercept) the calibration plot showed good concordance between the predicted and observed prevalence of severe baseline ACAS. The risk was overestimated in the highest decile of predicted risk (Figure 1). Discrimination and calibration were slightly worse in women compared with men (Table S3; Figures S4 through S7).

**Table 2.**
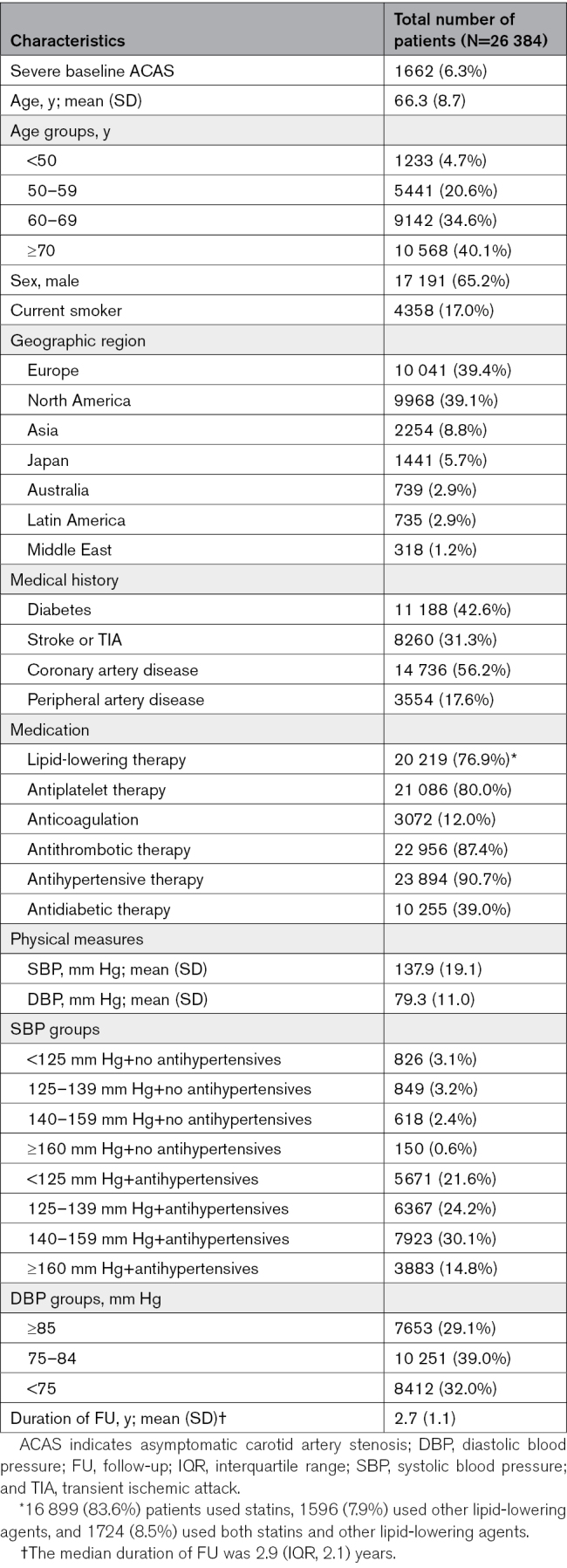
Baseline Characteristics of the Reduction of Atherothrombosis for Continued Health Registry

During the mean duration of 2.7 years of follow-up (70 799 patient-years for the outcome stroke and 69 949 for the outcome CVD), 1124 patients developed a stroke and 2484, CVD. Incidence rates of stroke and CVD were significantly higher in patients with severe baseline ACAS compared with no severe ACAS, with an incidence rate ratio of 1.97 (95% CI, 1.62–2.38) and an incidence rate ratio of 1.89 (95% CI, 1.66–2.15), respectively.

**Figure 2.**
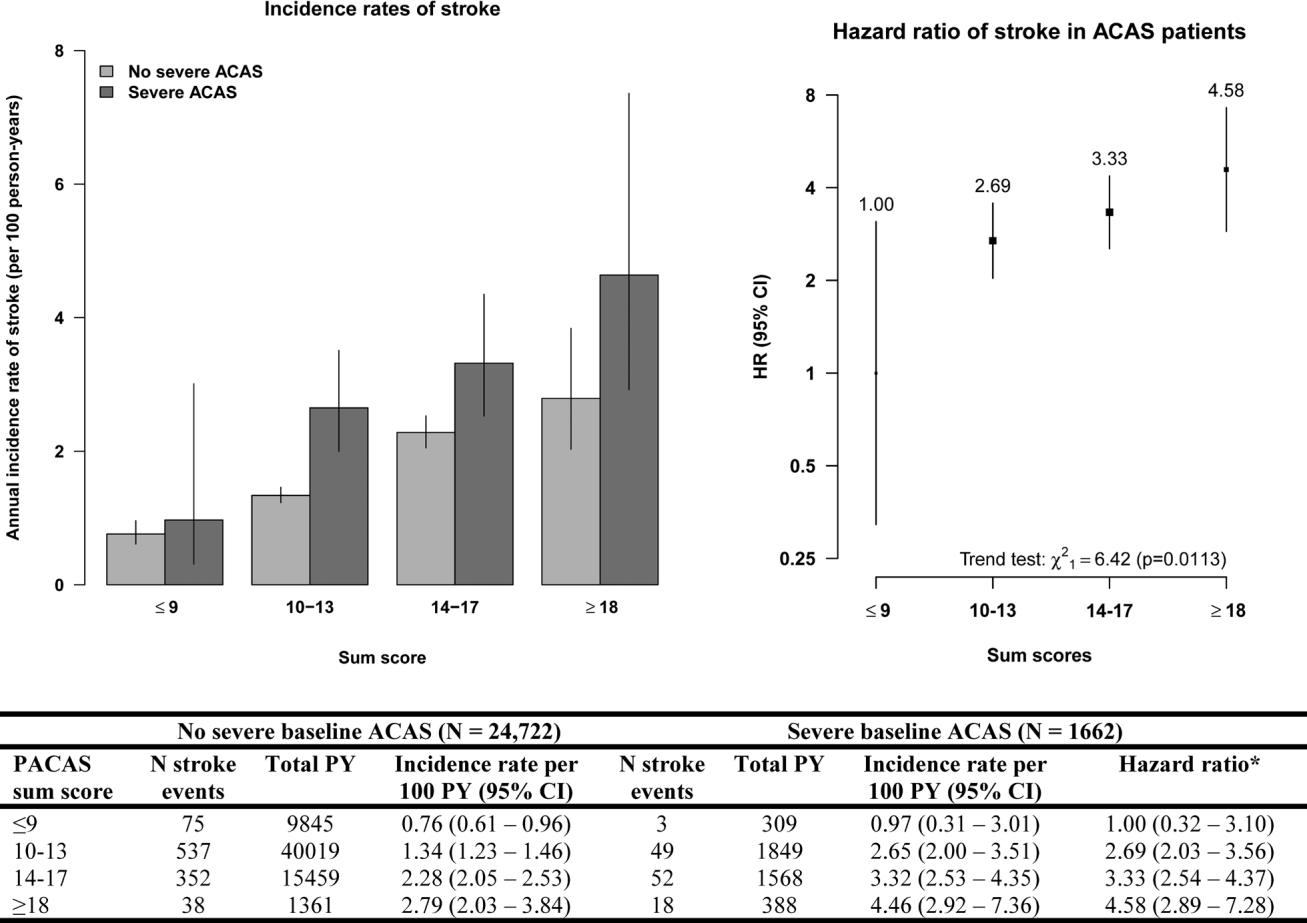
**Incidence rates and hazard ratios (HRs) of stroke during follow-up, by prevalence of asymptomatic carotid artery stenosis (PACAS) sum score.** The bar chart (**left**) shows the annual incidence rate of stroke (per 100 patient-years [PYs]) in patients with and without severe baseline asymptomatic carotid artery stenosis (ACAS) for each PACAS risk group. The figure (**right**) shows the HRs of stroke in patients with severe baseline ACAS. The vertical bars represent the 95% CIs in both figures. *We calculated the HRs of stroke in patients with severe baseline ACAS using group-specific CIs.

Incidence rates of stroke among ACAS patients rose with increasing PACAS sum score from 1.0 ([95% CI, 0.3–3.0] sum score, ≤9), 2.7 ([95% CI, 2.0–3.5] sum score, 10–13), and 3.3 ([95% CI, 2.5–4.4] sum score, 14–17) to 4.6 ([95% CI, 2.9–7.4] sum score, ≥18) per 100 patient-years (Figure 2). The hazard ratio of stroke was 2.69 (95% CI, 2.03–3.56) in patients with sum score of 10 to 13; 3.33 (95% CI, 2.54–4.37) in patients with sum score of 14 to 17, and 4.58 (95% CI, 2.89–7.28) in patients with sum score ≥18 compared with patients with sum score ≤9 (*P*_trend_<0.017; Figure 2).

**Figure 3.**
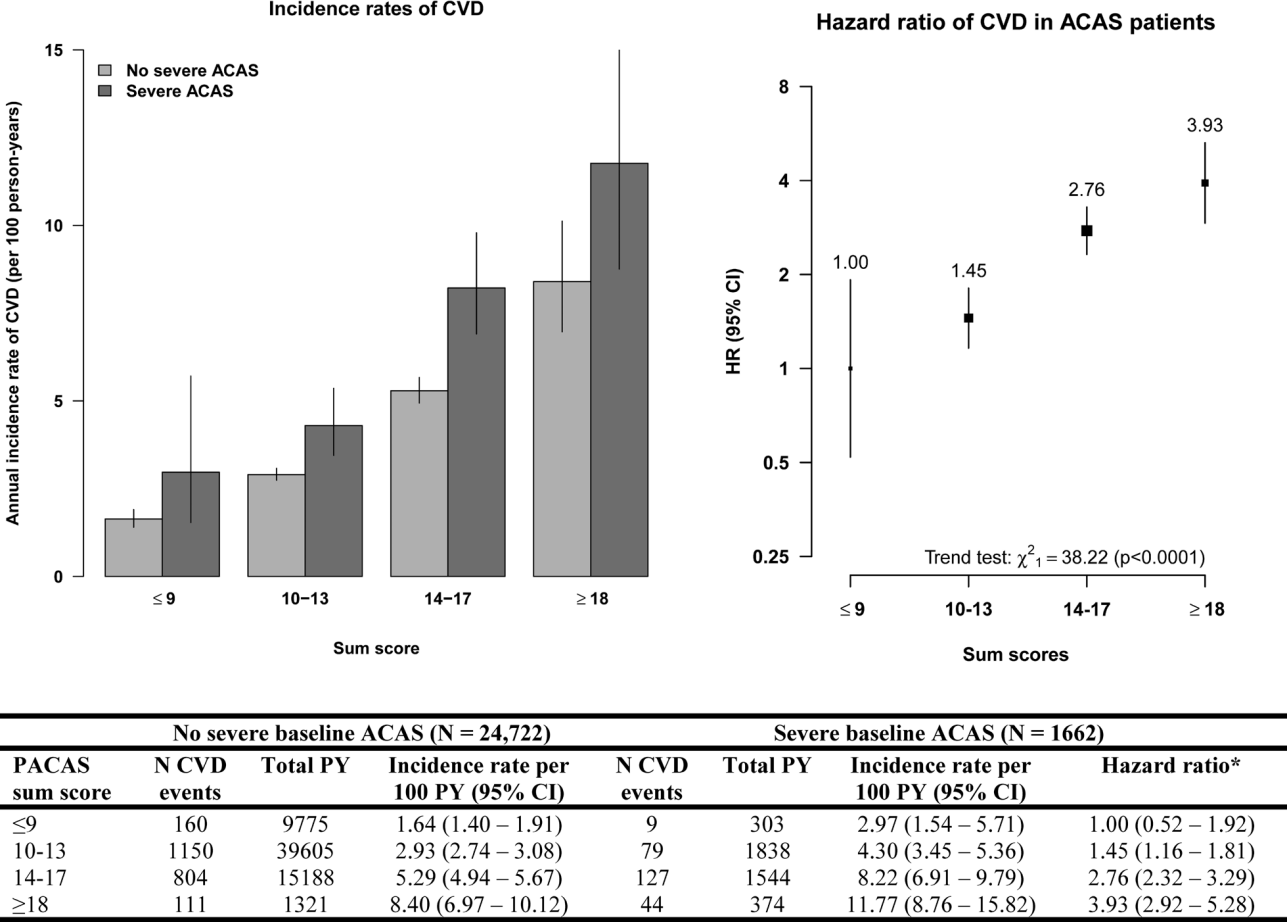
**Incidence rates and hazard ratios (HRs) of cardiovascular disease (CVD) during follow-up, by prevalence of asymptomatic carotid artery stenosis (PACAS) sum score.** The bar chart (**left**) shows the annual incidence rate of CVD (per 100 patient-years [PYs]) in patients with and without severe baseline asymptomatic carotid artery stenosis (ACAS) for each PACAS risk group. The figure (**right**) shows the HRs of CVD in patients with severe baseline ACAS. The vertical bars represent the 95% CIs in both figures. *We calculated the HRs of CVD in patients with severe baseline ACAS using group-specific CIs.

A similar increase in incidence rates of CVD among ACAS patients was found, with incidence rates of 3.0 ([95% CI, 1.5–5.7] sum score, ≤9), 4.3 ([95% CI, 3.4–5.4] sum score, 10–13), and 8.2 ([95% CI, 6.9–9.8] sum score, 14–17) to 11.8 ([95% CI, 8.8–15.8] sum score, ≥18) per 100 patient-years (Figure 3). The hazard ratio of CVD was 1.45 (95% CI, 1.16–1.81) in patients with sum score 10 to 13, 2.76 (95% CI, 2.32–3.29) in patients with sum score 14 to 17, and 3.93 (95% CI, 2.92–5.28) in patients with sum score ≥18 compared with patients with sum score ≤9 (*P*_trend_<0.0001; Figure 3).

Selective screening for severe ACAS in patients with PACAS sum scores of ≥14 showed a prevalence of 11.4%, resulting in an NNS of 9. This selective screening identified approximately half of patients with severe baseline ACAS by screening 27.7% of all patients (Table 3). Of all strokes and CVD events during follow-up that occurred in patients with severe ACAS, 56.6% and 64.9% occurred in ACAS patients with sum score of ≥14, respectively (Figure 2).

**Table 3.**
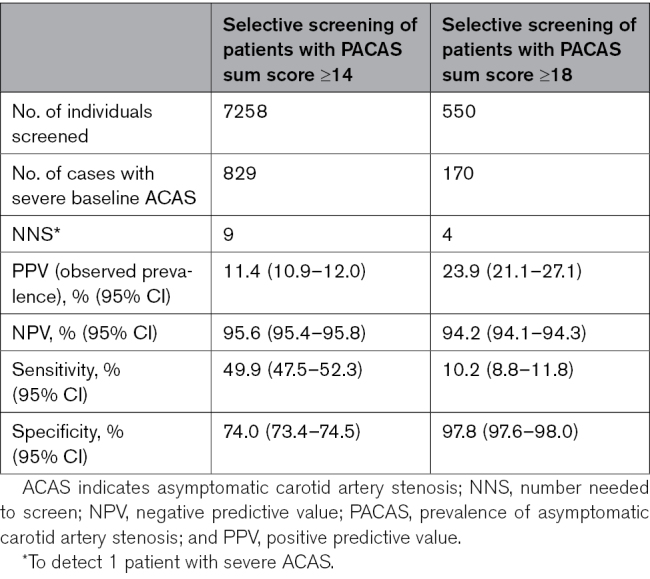
Screening Characteristics of PACAS in the Reduction of Atherothrombosis for Continued Health Registry

Selective screening for severe ACAS in patients with PACAS sum scores of ≥18 showed a prevalence of 23.9%, resulting in an NNS of 4. This selective screening identified 10.2% of patients with severe baseline ACAS by screening 2.1% of all patients (Table 3). Of all strokes and CVD events during follow-up that occurred in severe ACAS patients, 14.8% and 17.0% occurred in ACAS patients with sum score of ≥18, respectively (Figure 3).

## DISCUSSION

Our study showed reasonable external validity of the PACAS risk model to detect severe ACAS in a population with established atherothrombotic disease or ≥3 atherothrombotic risk factors. Selective screening of patients with sum scores of ≥14 (27.7% of all patients) identified approximately half of patients with severe baseline ACAS, resulting in an NNS of 9. Incidence rates of stroke and CVD during follow-up were significantly elevated in patients with higher PACAS sum scores, indicating that selective screening with the PACAS risk model can identify patients in whom the greatest risk reduction can be achieved.

The PACAS risk model is able to provide reliable predictions in different settings. The derivation study showed that the PACAS risk score could be used in preclinical populations of screened people with a low proportion of patients with CVD (stroke, 3.4%; coronary artery disease, 5.3%; and peripheral arterial disease, 3.1%).^10^ The current study shows that the PACAS risk model also provides reliable predictions in a clinical setting with a high proportion of patients with CVD (stroke or TIA, 31.3%; coronary artery disease, 56.2%; and peripheral arterial disease, 17.6%).

A previous large-scale trial that assessed population-level CVD screening by randomly inviting patients for noncontrast electrocardiography-gated computed tomography (for coronary artery calcium score, aneurysms, and atrial fibrillation), ankle-brachial blood pressure measurements (for peripheral arterial disease and hypertension), and a blood sample (diabetes and hypercholesterolemia) failed to show that cardiovascular risk management reduced all-cause mortality, but stroke was significantly reduced by 7%.^21^ This reinforces the need for new risk modifiers that can help reducing the burden of CVD in addition to conventional vascular risk factors.^22^ Different imaging parameters have been proposed, but widespread implementation is hampered due to unclear long-term benefit of early detection with subsequently initiated preventive treatment.^1^

Coronary artery calcium scoring has been recommended in the recent European Society of Cardiology guidelines for patients with predicted risks around decision thresholds but only in locoregional contexts because of availability and cost-effectiveness.^1^ Previous studies on carotid ultrasound using intima-media thickness of the (common) carotid artery only found a negligible improvement in predicting first CVD events.^23,24^ In contrast, our study suggests that velocity-based stenoses might potentially be a stronger risk modifier.

Carotid-specific interventions, such as carotid surgery and stenting, are also available.^25,26^ These interventions do not prevent all future strokes but approximately half the long-term risk of incidence of stroke.^25^ However, with declining risks of ipsilateral stroke over time (without intervention), these interventions should only be restricted to selected patients in whom the procedural risks are outweighed sufficiently.^3^ Previous studies showed comparable long-term outcomes after carotid surgery and stenting but higher procedural risks of carotid stenting.^27,28^ Risk models to select those patients at high risk of ipsilateral stroke under medical therapy that might warrant a carotid intervention have not been validated.^29,30^ Validated models will help identifying a subset of patients in whom there might be a benefit of a carotid procedure but also to refrain from unnecessary procedures in patients with low risks of ipsilateral stroke. The latest Society for Vascular Surgery and European Society for Vascular Surgery guidelines for the management of carotid disease have acknowledged a validated risk model as a research priority.^4,5^ If clear indications for carotid interventions in patients with ACAS are established, this will reinforce the need for selective screening.

The present study has several strengths. A large cohort of patients with documented symptomatic CVD or multiple risk factors was used. All predictors of the PACAS risk model were available in the REACH registry, except HDL to calculate the total cholesterol/HDL cholesterol ratio. We proposed 2 possible cutoffs for selection of patients for selective screening based on the sum score of the PACAS risk model. We updated the PACAS risk equation with adjustment for differences in the derivation cohort and the REACH registry because the overall prevalence of severe baseline ACAS in the derivation cohort was 0.3% compared with 6.3% in the REACH registry.^10^ Missing data were limited (except for peripheral arterial disease), and we used multiple imputation to handle missing data. The present study also has several limitations. The status of the carotid arteries was reported as unknown in a substantial part of patients. A previous publication showed that these patients differed in risk factor profile from patients with severe baseline ACAS.^31^ The presence of severe stenosis was determined by duplex ultrasound or digital subtraction angiography and reported by treating physicians introducing potential differences in determining the degree of stenosis between centers. History of stroke or TIA and stroke outcomes in the REACH registry were not classified according to location (ipsilateral, contralateral, or vertebrobasilar). Symptomatic status of the carotid arteries was not recorded, but the research protocol of the REACH registry prescribed that patients with a history of stroke or TIA are not considered asymptomatic. We excluded all patients with a carotid intervention at baseline and censored patients at the time of a carotid intervention during follow-up to avoid an underestimation of the risk of stroke under medical therapy. We were not able to determine geographic variation due to the limited number of patients with severe ACAS. Recruitment of the REACH registry ended 2 decades ago, and the duration of follow-up was relatively short.

Future studies with longer duration of follow-up should confirm the incidence rates of stroke and CVD in ACAS patients with high PACAS sum scores. Imaging parameters of plaque vulnerability could provide more precise predictions of long-term outcomes and a higher sensitivity of the PACAS risk model to predict stenoses that are associated with stroke and CVD. In addition, social determinants of health might improve the prediction toward socially deprived patients. The number of stroke and CVD events that could be prevented by using more intensive preventive therapy in patients with detected ACAS should be determined to develop a cost-effective selective screening program. Interventional studies might use the PACAS risk score for patient selection to tailor preventive therapies to sum scores, in a similar way as the CHA_2_DS_2_-VASc risk score is used to select patients with atrial fibrillation for anticoagulant therapy. Carotid stenosis may also have an incremental benefit as a predictor in cardiovascular risk stratification with established risk models such as the SMART2 (Secondary Manifestations of Arterial Disease) or SMART-REACH (Secondary Manifestations of Arterial Disease—Reduction of Atherothrombosis for Continued Health) model,^32,33^ Future studies should also determine the utility of the PACAS risk model or novel risk models to predict ipsilateral stroke, thereby identifying a subset of patients with ACAS in whom there may be a benefit of carotid interventions. Biobank studies with data on imaging of the carotid arteries should, therefore, consider including velocity-based stenoses.^34^

In conclusion, The PACAS risk model can reliably identify patients at high risk of severe ACAS in a population of patients with established atherothrombotic disease or ≥3 atherothrombotic risk factors. It can, therefore, be used for selective screening to reduce the NNS considerably. And since incidence rates of stroke and CVD during follow-up were significantly elevated in patients with higher PACAS sum scores, selective screening using the PACAS risk model will detect patients with ACAS at high risk of major adverse cardiovascular events. In these patients, the greatest risk reduction of future stroke or CVD can be achieved by improving prevention strategies.

## ARTICLE INFORMATION

### Acknowledgments

We are grateful to Paul Sherliker (Nuffield Department of Population Health, University of Oxford) for help creating the figures.

### Sources of Funding

None.

### Disclosures

Dr Bhatt discloses the following relationships: advisory board: Angiowave, Bayer, Boehringer Ingelheim, CellProthera, Cereno Scientific, Elsevier Practice Update Cardiology, High Enroll, Janssen, Level Ex, McKinsey, Medscape Cardiology, Merck, MyoKardia, NirvaMed, Novo Nordisk, PhaseBio, PLx Pharma, and Stasys; board of directors: American Heart Association New York City, Angiowave (stock options), Bristol Myers Squibb (stock), DRS.LINQ (stock options), and High Enroll (stock); consultant: Broadview Ventures, Hims, SFJ, and Youngene; data monitoring committees: Acesion Pharma, Assistance Publique-Hôpitaux de Paris, Baim Institute for Clinical Research (formerly Harvard Clinical Research Institute, for the PORTICO trial [Portopulmonary Hypertension Treatment With Macitentan: A Randomized Clinical Trial], funded by St. Jude Medical, now Abbott), Boston Scientific (chair: the PEITHO trial [Pulmonary Embolism Thrombolysis]), Cleveland Clinic, Contego Medical (chair: PERFORMANCE 2 [Protection Against Emboli During Carotid Artery Stenting Using the Neuroguard IEP System]), Duke Clinical Research Institute, Mayo Clinic, Mount Sinai School of Medicine (for the ENVISAGE trial, funded by Daiichi Sankyo; for the ABILITY-DM trial, funded by Concept Medical; for ALLAY-HF [Safety and Efficacy of the Alleviant System for No-Implant Interatrial Shunt Creation in Patients With Chronic Heart Failure], funded by Alleviant Medical), Novartis, Population Health Research Institute, and Rutgers University (for the National Institutes of Health-funded MINT trial [Myocardial Ischemia and Transfusion]); honoraria: American College of Cardiology (senior associate editor: Clinical Trials and News, ACC.org; chair: ACC Accreditation Oversight Committee), Arnold and Porter law firm (work related to Sanofi/Bristol Myers Squibb clopidogrel litigation), Baim Institute for Clinical Research (formerly Harvard Clinical Research Institute; the RE-DUAL PCI [Evaluation of Dual Therapy With Dabigatran vs Triple Therapy With Warfarin in Patients With AF That Undergo a PCI With Stenting] Clinical Trial Steering Committee funded by Boehringer Ingelheim; AEGIS-II [Study to Investigate CSL112 in Subjects With Acute Coronary Syndrome] Executive Committee funded by CSL Behring), Belvoir Publications (editor-in-chief: Harvard Heart Letter), Canadian Medical and Surgical Knowledge Translation Research Group (clinical trial steering committees), CSL Behring (AHA lecture), Cowen and Company, Duke Clinical Research Institute (clinical trial steering committees, including for the PRONOUNCE trial [A Trial Comparing Cardiovascular Safety of Degarelix Versus Leuprolide in Patients With Advanced Prostate Cancer and Cardiovascular Disease], funded by Ferring Pharmaceuticals), HMP Global (editor-in-chief: *Journal of Invasive Cardiology*), *Journal of the American College of Cardiology* (guest editor, associate editor), K2P (co-chair: interdisciplinary curriculum), Level Ex, Medtelligence/ReachMD (Continuing Medical Education [CME] steering committees), MJH Life Sciences, Oakstone CME (course director: Comprehensive Review of Interventional Cardiology), Piper Sandler, Population Health Research Institute (for the COMPASS [Cardiovascular Outcomes for People Using Anticoagulation Strategies] Operations Committee, Publications Committee, Steering Committee, and USA national coleader, funded by Bayer), WebMD (CME steering committees), and Wiley (steering committee); other: Clinical Cardiology (deputy editor); patent: Sotagliflozin (named on a patent for sotagliflozin assigned to Brigham and Women’s Hospital that assigned to Lexicon; neither he nor Brigham and Women’s Hospital receive any income from this patent); research funding: Abbott, Acesion Pharma, Afimmune, Aker Biomarine, Alnylam, Amarin, Amgen, AstraZeneca, Bayer, Beren, Boehringer Ingelheim, Boston Scientific, Bristol Myers Squibb, Cardax, CellProthera, Cereno Scientific, Chiesi, CinCor, Cleerly, CSL Behring, Eisai, Ethicon, Faraday Pharmaceuticals, Ferring Pharmaceuticals, Forest Laboratories, Fractyl, Garmin, HLS Therapeutics, Idorsia, Ironwood, Ischemix, Janssen, Javelin, Lexicon, Lilly, Medtronic, Merck, Moderna, MyoKardia, NirvaMed, Novartis, Novo Nordisk, Otsuka, Owkin, Pfizer, PhaseBio, PLx Pharma, Recardio, Regeneron, Reid Hoffman Foundation, Roche, Sanofi, Stasys, Synaptic, The Medicines Company, Youngene, and 89Bio; royalties: Elsevier (editor: Braunwald’s Heart Disease); site coinvestigator: Abbott, Biotronik, Boston Scientific, CSI, Endotronix, St. Jude Medical (now Abbott), Philips, SpectraWAVE, Svelte, and Vascular Solutions; trustee: American College of Cardiology; and unfunded research: FlowCo. Dr Steg discloses the following relationships: employment: Assistance Publique-Hôpitaux de Paris and Université Paris-Cité; research grants: Bayer; consulting: Amarin, Amgen, AstraZeneca, Bayer, Bristol Myers Squibb, Boehringer Ingelheim, Janssen, Pfizer, PhaseBio, Novartis, NovoNordisk, Regeneron, Sanofi, and Servier; and patent issued as coinventor of use of alirocumab to reduce cardiovascular risk (patent assigned to Sanofi). The other authors report no conflicts.

### Supplemental Material

Tables S1–S3

Figures S1–S7

## Supplementary Material
