# Supplementary material for: Prediction of Severe Baseline Asymptomatic Carotid Stenosis and Subsequent Risk of Stroke and Cardiovascular Disease
Source: Stroke. 2024 Sep 25;55(11):2632–40. doi: 10.1161/STROKEAHA.123.046894 (PMC11518973; doi:10.1161/STROKEAHA.123.046894)
Supplement: Supplementary file 1 [file str-55-2632-s001.pdf]

# Prediction of severe baseline asymptomatic carotid stenosis and subsequent risk of stroke and cardiovascular disease

## Supplemental Material

## Table S1. Percentage of missing values for each predictor

| Characteristics | Percentage of missing values |
|---|---|
| Age | 0 |
| Sex | 0.04 |
| Current smoker | 2.6 |
| Diabetes | 0.4 |
| History of prior stroke or TIA | 0 |
| Coronary Artery Disease | 0.6 |
| Peripheral Artery Disease | 23.5 |
| Lipid lowering therapy | 0.3 |
| Antiplatelet therapy | 0.1 |
| Anticoagulation | 2.9 |
| Antithrombotic therapy | 0.4 |
| Antihypertensive drugs | 0.1 |
| Antidiabetic therapy | 0.2 |
| SBP | 0.2 |
| DBP | 0.3 |

DBP, diastolic blood pressure; SBP, systolic blood pressure; TIA, transient ischemic attack.

# Table S2. TRIPOD checklist

| Section/Topic | Item | Checklist Item | Page |
|---|---|---|---|
| **Title and abstract** | | | |
| **Title** | 1 | Identify the study as developing and/or validating a multivariable prediction model, the target population, and the outcome to be predicted. | 1 |
| **Abstract** | 2 | Provide a summary of objectives, study design, setting, participants, sample size, predictors, outcome, statistical analysis, results, and conclusions. | 2-3 |
| **Introduction** | | | |
| **Background and objectives** | 3a | Explain the medical context (including whether diagnostic or prognostic) and rationale for developing or validating the multivariable prediction model, including references to existing models. | 4-5 |
| | 3b | Specify the objectives, including whether the study describes the development or validation of the model or both. | 4-5 |
| **Methods** | | | |
| **Source of data** | 4a | Describe the study design or source of data (e.g., randomized trial, cohort, or registry data), separately for the development and validation data sets, if applicable. | 6 |
| | 4b | Specify the key study dates, including start of accrual; end of accrual; and, if applicable, end of follow-up. | 6 |
| **Participants** | 5a | Specify key elements of the study setting (e.g., primary care, secondary care, general population) including number and location of centres. | 6 |
| | 5b | Describe eligibility criteria for participants. | 6 |
| | 5c | Give details of treatments received, if relevant. | 6 |
| **Outcome** | 6a | Clearly define the outcome that is predicted by the prediction model, including how and when assessed. | 7 |
| | 6b | Report any actions to blind assessment of the outcome to be predicted. | NA |
| **Predictors** | 7a | Clearly define all predictors used in developing or validating the multivariable prediction model, including how and when they were measured. | 7 |
| | 7b | Report any actions to blind assessment of predictors for the outcome and other predictors. | 7 |
| **Sample size** | 8 | Explain how the study size was arrived at. | Fig 1 |
| **Missing data** | 9 | Describe how missing data were handled (e.g., complete-case analysis, single imputation, multiple imputation) with details of any imputation method. | 8 |
| **Statistical analysis methods** | 10c | For validation, describe how the predictions were calculated. | 8 |
| | 10d | Specify all measures used to assess model performance and, if relevant, to compare multiple models. | NA |
| | 10e | Describe any model updating (e.g., recalibration) arising from the validation, if done. | 8 |
| **Risk groups** | 11 | Provide details on how risk groups were created, if done. | 8 |
| **Development vs. validation** | 12 | For validation, identify any differences from the development data in setting, eligibility criteria, outcome, and predictors. | 8 |
| **Results** | | | |
| **Participants** | 13a | Describe the flow of participants through the study, including the number of participants with and without the outcome and, if applicable, a summary of the follow-up time. A diagram may be helpful. | Fig 1 |
| | 13b | Describe the characteristics of the participants (basic demographics, clinical features, available predictors), including the number of participants with missing data for predictors and outcome. | Table 1 & Suppl table 2 |
| | 13c | For validation, show a comparison with the development data of the distribution of important variables (demographics, predictors and outcome). | NA |
| **Model performance** | 16 | Report performance measures (with Cis) for the prediction model. | 9 |
| **Model-updating** | 17 | If done, report the results from any model updating (i.e., model specification, model performance). | 9 |
| **Discussion** | | | |
| **Limitations** | 18 | Discuss any limitations of the study (such as nonrepresentative sample, few events per predictor, missing data). | 12 |
| **Interpretation** | 19a | For validation, discuss the results with reference to performance in the development data, and any other validation data. | 11 |
| | 19b | Give an overall interpretation of the results, considering objectives, limitations, results from similar studies, and other relevant evidence. | 11 |
| **Implications** | 20 | Discuss the potential clinical use of the model and implications for future research. | 12 |

| | | | |
|---|---|---|---|
| **Other information** | | | |
| **Supplementary information** | 21 | Provide information about the availability of supplementary resources, such as study protocol, Web calculator, and data sets. | 13 |
| **Funding** | 22 | Give the source of funding and the role of the funders for the present study. | 13 |

# Table S3. Discrimination values in men and women

| AUROC | Men | Women |
|---|---|---|
| Risk score | 0.67 (95% CI 0.66-0.69) | 0.62 (95% CI 0.59-0.64) |
| Risk equation | 0.69 (95% CI 0.65-0.74) | 0.64 (95% CI 0.58-0.70) |

The AUROC curves were calculated in the imputed data sets with 17,191 included men and 9183 women.
CI, confidence interval

## Figure S1. Flowchart of included patients of the REACH dataset

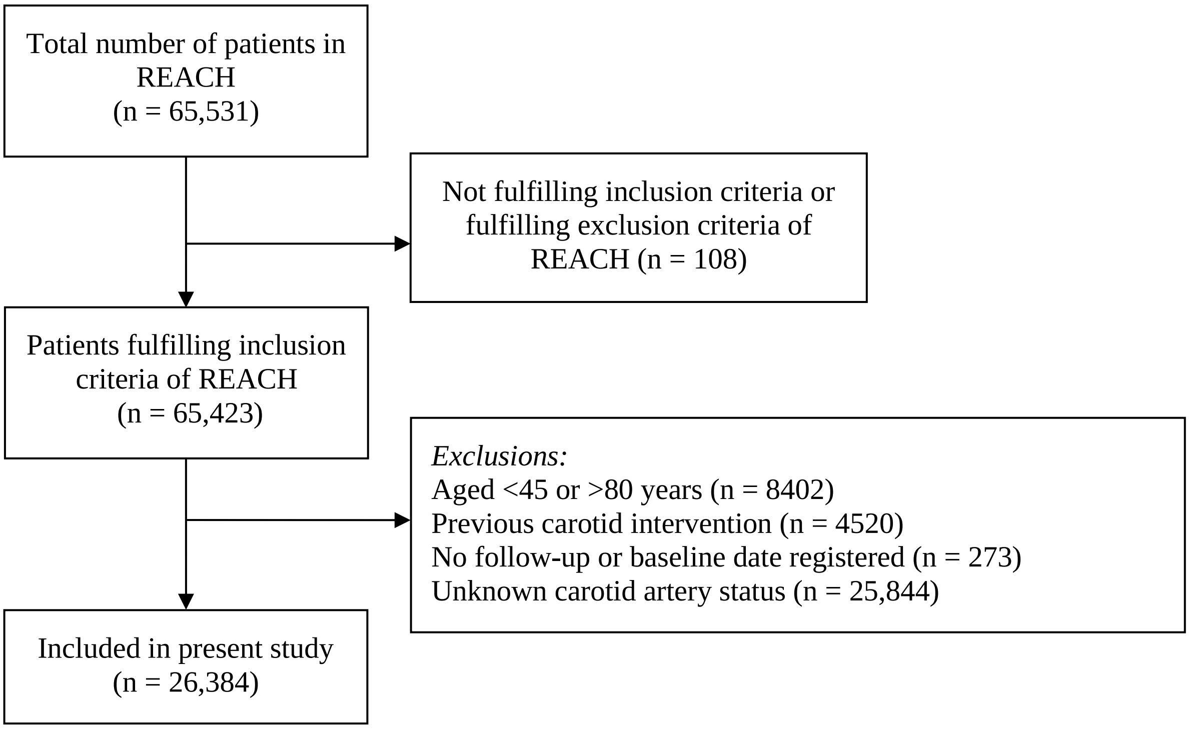

# Figure S2. Distribution of PACAS sum scores

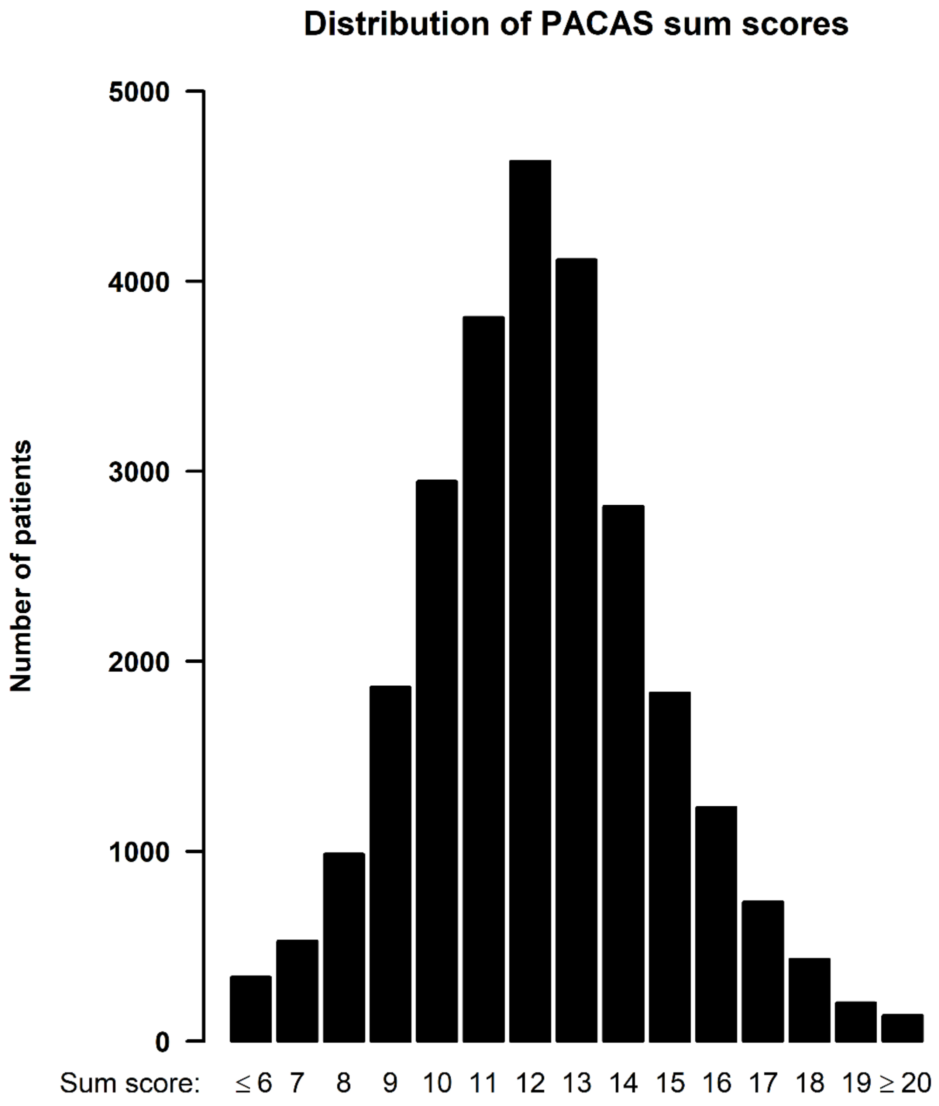

# Figure S3. Calibration plot before recalibration in all patients

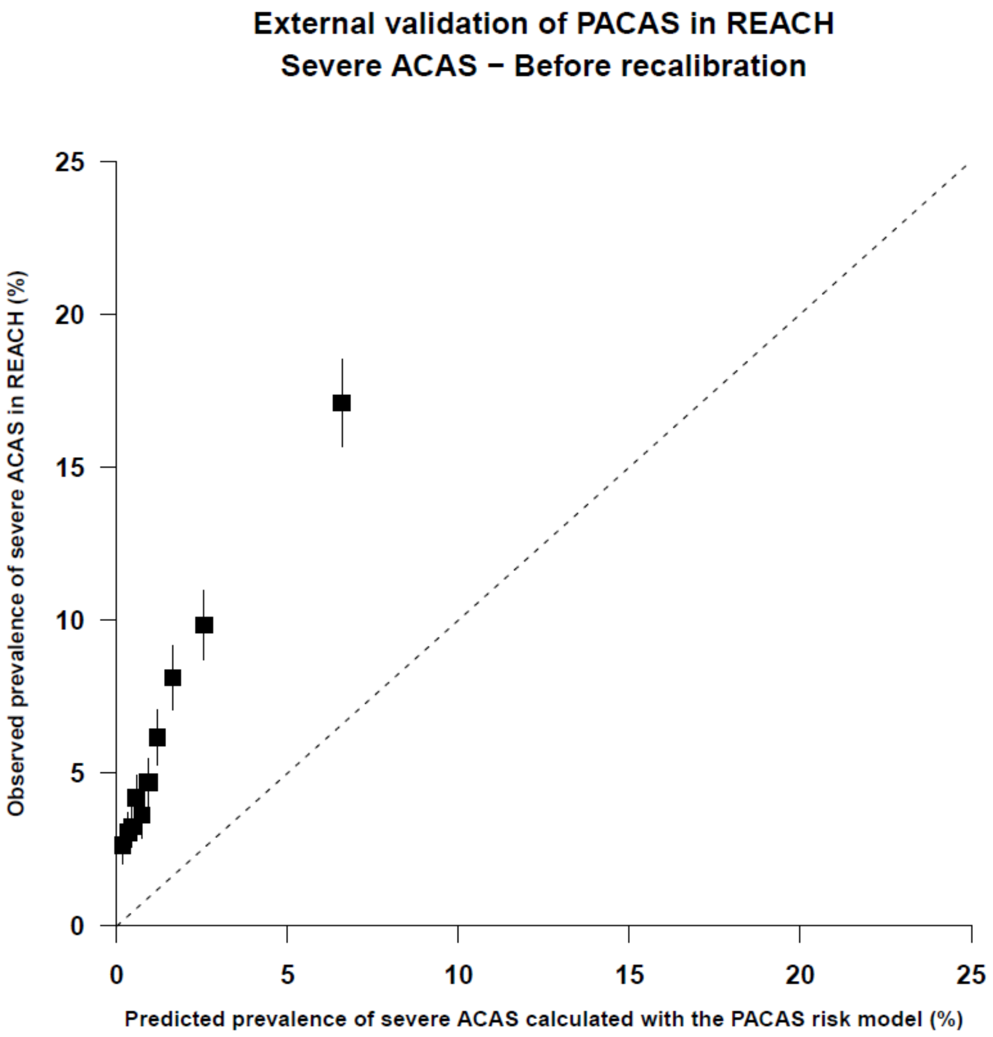


| | | | | | | | | | | |
|---|---|---|---|---|---|---|---|---|---|---|
| **Total number of patients** | 2504 | 2691 | 2670 | 2653 | 2265 | 2953 | 2707 | 2635 | 2655 | 2652 |
| **Number of patients with severe baseline ACAS** | 73 | 73 | 95 | 105 | 85 | 143 | 166 | 192 | 281 | 449 |
| **Observed prevalence in REACH** | 2.92 | 2.71 | 3.56 | 3.96 | 3.75 | 4.84 | 6.13 | 7.29 | 10.58 | 16.93 |
| **Predicted prevalence estimated with PACAS** | 0.17 | 0.33 | 0.45 | 0.58 | 0.74 | 0.92 | 1.18 | 1.64 | 2.55 | 6.60 |

The calibration plot showing the mean predicted risk against the observed risk of severe baseline ACAS across deciles of predicted risk. The boxes represent the mean predicted risk for each decile and the vertical lines represent the 95% confidence intervals. The dotted diagonal line indicates perfect calibration. Boxes above the diagonal line indicate underestimation of risk and below the diagonal line overestimation of risk.

# Figure S4. Calibration plot before recalibration in men

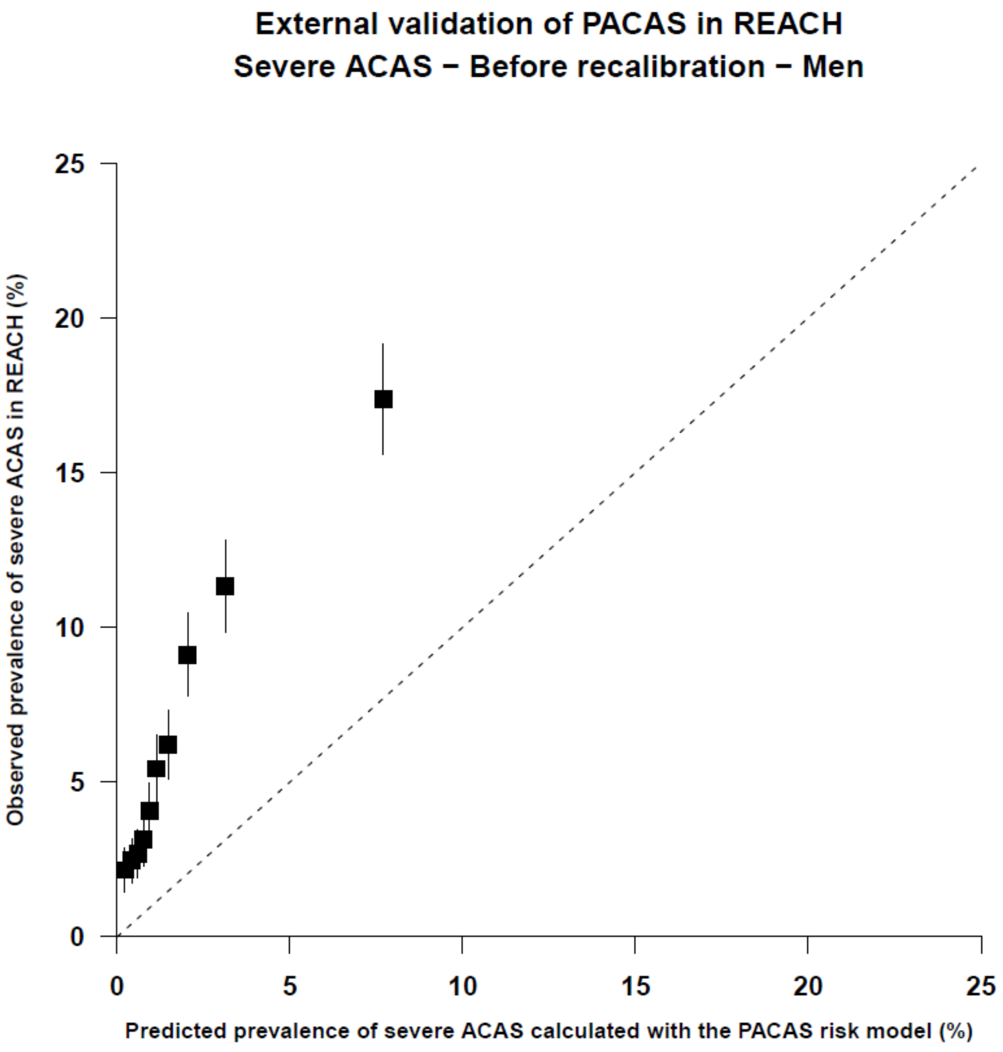


| | | | | | | | | | | |
|---|---|---|---|---|---|---|---|---|---|---|
| **Total number of men** | 1582 | 1823 | 1710 | 1691 | 1773 | 1640 | 1771 | 1732 | 1746 | 1723 |
| **Number of men with severe baseline ACAS** | 34 | 45 | 46 | 53 | 72 | 89 | 110 | 158 | 198 | 299 |
| **Observed prevalence in REACH** | 2.15 | 2.47 | 2.69 | 3.13 | 4.06 | 5.43 | 6.21 | 9.12 | 11.34 | 17.35 |
| **Predicted prevalence estimated with PACAS** | 0.23 | 0.42 | 0.59 | 0.77 | 0.94 | 1.14 | 1.48 | 2.05 | 3.14 | 7.71 |

The calibration plot showing the mean predicted risk against the observed risk of severe baseline ACAS across deciles of predicted risk. The boxes represent the mean predicted risk for each decile and the vertical lines represent the 95% confidence intervals. The dotted diagonal line indicates perfect calibration. Boxes above the diagonal line indicate underestimation of risk and below the diagonal line overestimation of risk.

# Figure S5. Calibration plot after recalibration (updating the intercept) in men

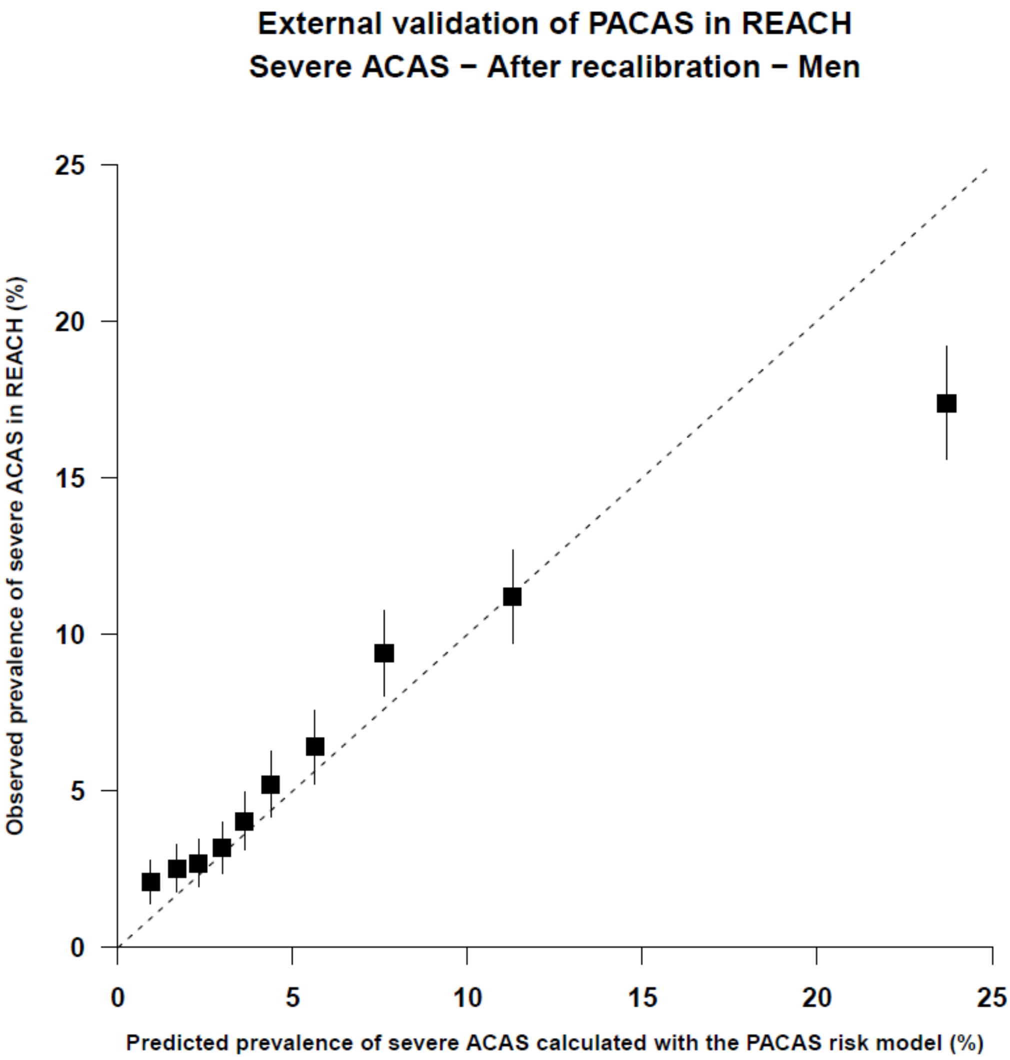


| | | | | | | | | | | |
|---|---|---|---|---|---|---|---|---|---|---|
| **Total number of men** | 1716 | 1699 | 1705 | 1752 | 1707 | 1729 | 1703 | 1736 | 1724 | 1720 |
| **Number of men with severe baseline ACAS** | 36 | 43 | 46 | 56 | 69 | 90 | 109 | 163 | 193 | 299 |
| **Observed prevalence in REACH** | 2.10 | 2.53 | 2.70 | 3.20 | 4.04 | 5.21 | 6.40 | 9.39 | 11.19 | 17.38 |
| **Predicted prevalence estimated with PACAS** | 0.93 | 1.69 | 2.30 | 2.97 | 3.62 | 4.38 | 5.63 | 7.62 | 11.29 | 23.70 |

The calibration plot showing the mean predicted risk against the observed risk of severe baseline ACAS across deciles of predicted risk. The boxes represent the mean predicted risk for each decile and the vertical lines represent the 95% confidence intervals. The dotted diagonal line indicates perfect calibration. Boxes above the diagonal line indicate underestimation of risk and below the diagonal line overestimation of risk.

# Figure S6. Calibration plot before recalibration in women

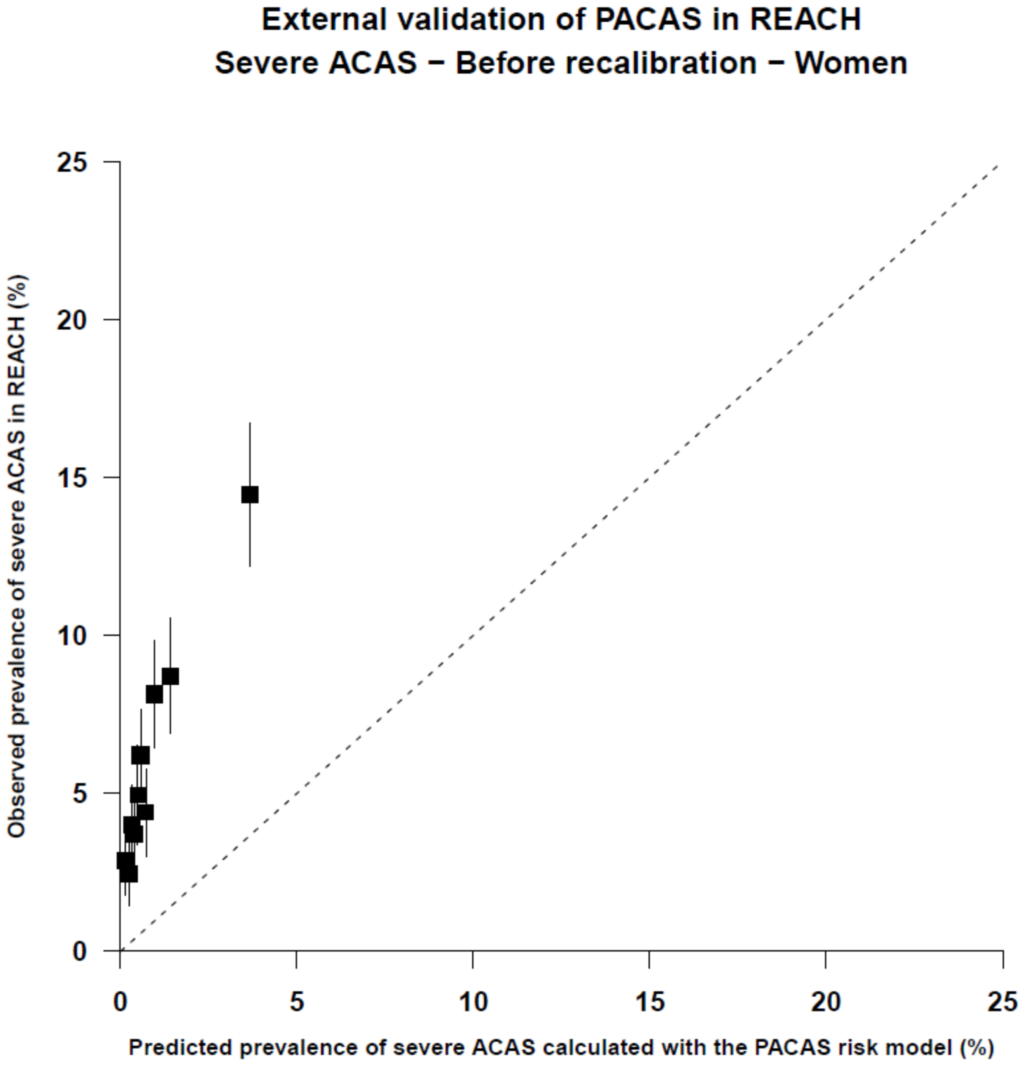


| | | | | | | | | | | |
|---|---|---|---|---|---|---|---|---|---|---|
| **Total number of women** | 910 | 897 | 901 | 947 | 728 | 1125 | 841 | 996 | 918 | 920 |
| **Number of women with severe baseline ACAS** | 26 | 22 | 36 | 35 | 36 | 70 | 37 | 81 | 80 | 133 |
| **Observed prevalence in REACH** | 2.86 | 2.45 | 4.00 | 3.70 | 4.95 | 6.22 | 4.40 | 8.13 | 8.71 | 14.46 |
| **Predicted prevalence estimated with PACAS** | 0.13 | 0.24 | 0.31 | 0.40 | 0.48 | 0.57 | 0.73 | 0.96 | 1.43 | 3.67 |

The calibration plot showing the mean predicted risk against the observed risk of severe baseline ACAS across deciles of predicted risk. The boxes represent the mean predicted risk for each decile and the vertical lines represent the 95% confidence intervals. The dotted diagonal line indicates perfect calibration. Boxes above the diagonal line indicate underestimation of risk and below the diagonal line overestimation of risk.

# Figure S7. Calibration plot after recalibration (updating the intercept) in women

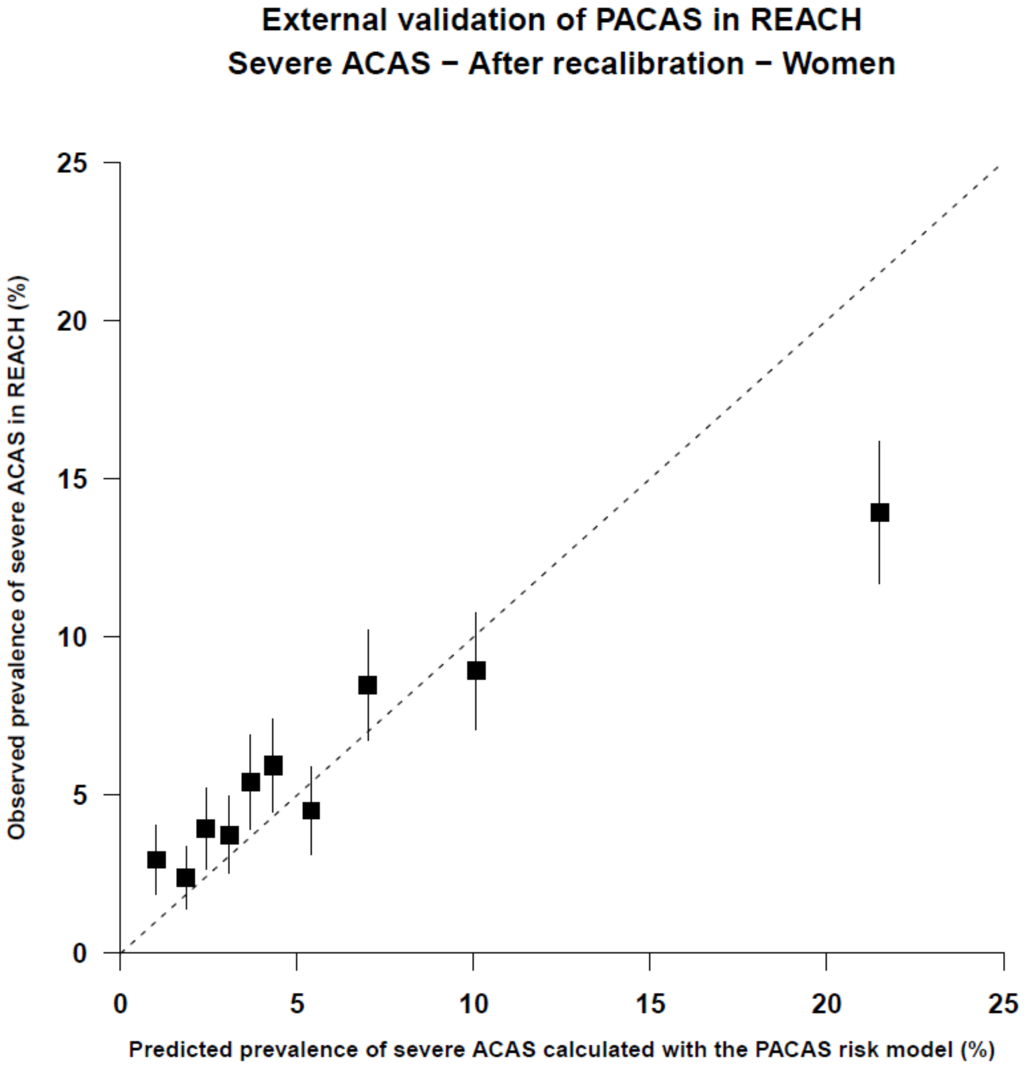


| | | | | | | | | | | |
|---|---|---|---|---|---|---|---|---|---|---|
| **Total number of women** | 913 | 919 | 888 | 937 | 869 | 977 | 863 | 980 | 918 | 919 |
| **Number of women with severe baseline ACAS** | 27 | 22 | 35 | 35 | 47 | 58 | 39 | 83 | 82 | 128 |
| **Observed prevalence in REACH** | 2.96 | 2.39 | 3.94 | 3.74 | 5.41 | 5.94 | 4.52 | 8.47 | 8.93 | 13.93 |
| **Predicted prevalence estimated with PACAS** | 1.02 | 1.85 | 2.42 | 3.08 | 3.69 | 4.32 | 5.40 | 7.00 | 10.07 | 21.49 |

The calibration plot showing the mean predicted risk against the observed risk of severe baseline ACAS across deciles of predicted risk. The boxes represent the mean predicted risk for each decile and the vertical lines represent the 95% confidence intervals. The dotted diagonal line indicates perfect calibration. Boxes above the diagonal line indicate underestimation of risk and below the diagonal line overestimation of risk.
